# Comparing growth in surface and cave morphs of the species *Astyanax mexicanus*: insights from scales

**DOI:** 10.1186/s13227-017-0086-6

**Published:** 2017-12-01

**Authors:** Victor Simon, Romain Elleboode, Kélig Mahé, Laurent Legendre, Patricia Ornelas-Garcia, Luis Espinasa, Sylvie Rétaux

**Affiliations:** 1 Paris-Saclay Institute of Neuroscience, CNRS UMR9197, Avenue de la terrasse, 91198 Gif-sur-Yvette, France; 2 Université Paris Sud and Paris-Saclay, Orsay, France; 3 IFREMER, Fisheries Laboratory, Sclerochronology Centre, 150 quai Gambetta, 62321 Boulogne-sur-Mer, France; 4 UMS AMAGEN, CNRS, INRA, Université Paris-Saclay, Gif-sur-Yvette, France; 5 Department of Zoology, Universidad Nacional Autónoma de México, Mexico City, Mexico; 6 School of Science, Marist College, 3399 North Rd, Poughkeepsie, NY 12601 USA

**Keywords:** Cave, Fish, Size, Age, Scales, Growth, Food, Comparative biology, Life history traits

## Abstract

**Background:**

Life in the darkness of caves is accompanied, throughout phyla, by striking phenotypic changes including the loss or severe reduction in eyes and pigmentation. On the other hand, cave animals have undergone constructive changes, thought to be adaptive, to survive in this extreme environment. The present study addresses the question of the evolution of growth in caves, taking advantage of the comparison between the river-dwelling and the cave-dwelling morphs of the Mexican tetra, *Astyanax mexicanus*.

**Results:**

A sclerochronology approach was undertaken to document the growth of the species in these two very distinct habitats. Scales from 158 wild *Astyanax mexicanus* specimens were analyzed from three caves (Pachón, Tinaja and Subterráneo) and two rivers (Rio Gallinas and Arroyo Lagarto) in San Luis Potosi and Tamaulipas, Mexico. A 10–13% reduction in scales size was observed in the cave morphs compared to the surface morphs. Age could be reliably inferred from annual growth increments on the scales from the two morphs of the species. Further comparisons with growth curves in laboratory conditions, obtained using the von Bertalanffy growth model, were also performed. In the wild and in the laboratory, cavefish originating from the Pachón cave reached smaller sizes than surface fish from three different locations: Rio Gallinas and Arroyo Lagarto (wild sampling) and Texas (laboratory population), respectively. Wild Pachón cavefish also seemed to grow to smaller sizes than the two other wild cavefish populations studied, Tinaja and Subterráneo. Finally, growth in the laboratory was faster than in the wild, particularly in the two first years of life.

**Conclusions:**

These data suggest that cavefish originating from the Pachón cave are subjected to an intrinsic limitation of their final size, which is at least in part independent from energy/food availability. This growth limitation may be an advantageous way of limiting energy expenditure and food needs in the cave environment. Moreover, growth regulation evolved differently in independently evolved cave populations. These results are discussed with regard to the sources of energy or general ecological conditions present in caves, and to the differences in behavior or feeding skills known in cavefish.

## Background

**Fig. 1:**
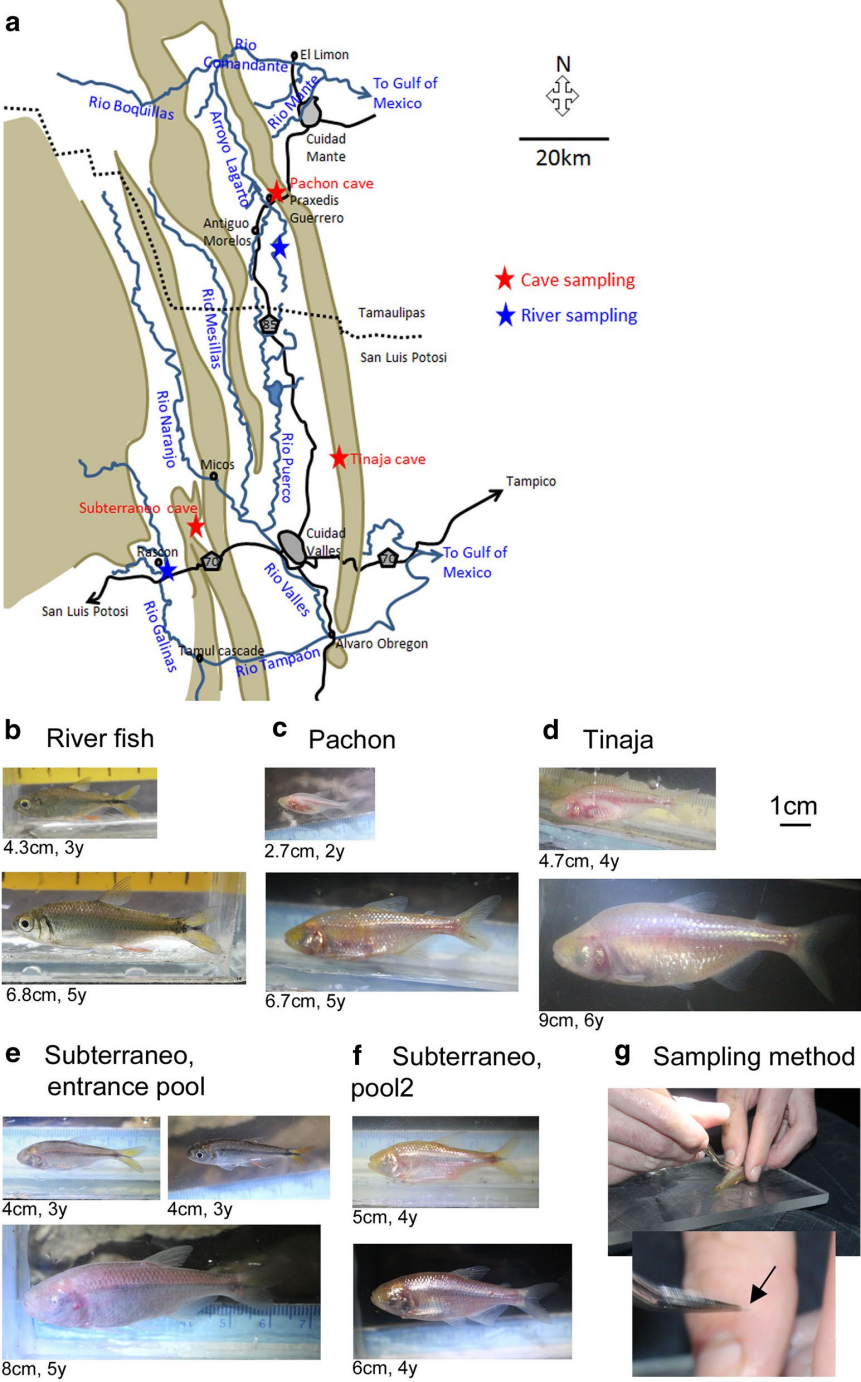
Sampling *Astyanax mexicanus* in the wild. **a** Map, with cave and surface (river) sampling points indicated as red and blue stars, respectively. Mountains are brown, rivers are blue, and roads are black. City names and state names are indicated. **b**–**f** Representative specimens caught in the indicated locations. Total lengths and ages are indicated. All photographs are at the same scale. Scale bar: 1 cm. **g** Sampling procedure

The characiform fish *Astyanax mexicanus* comes in two forms: one “normal” surface-dwelling morph, which inhabits the rivers of Mexico and the South of the USA, and blind and depigmented cave-dwelling morphs which are endemic to the caves of the Sierra de El Abra, Sierra de Guatemala and Sierra de Colmena in Mexico. Today, there are 29 described caves hosting troglomorphic *A. mexicanus* populations in this region [1]. Even though they are strikingly morphologically distinct from their surface-dwelling counterparts (see Fig. 1), all cave populations that have been tested so far are inter-fertile with surface fish and among themselves with a fertile progeny, indicating that they are conspecific. The *A. mexicanus* cavefish/surface fish model system is therefore increasingly used in evolutionary studies to address the developmental, genetic or genomic mechanisms of morphological evolution and behavioral adaptation [2–6].

The total and permanent darkness of caves is often considered as an extreme environment. Finding food and mates in the absence of vision is indeed the main challenge that cavefish and other cave animals have to solve. Moreover, due to the absence of photoautotrophic production, caves are frequently considered as food-poor and energy-poor. But depending on the cave location and its topography, quantities of carbon fluxes can sometimes be in the range of those reported for surface streams [7]. The sources of energy in the subterranean habitats are mainly threefold: (1) external sources, such as those from animals that enter the caves and either deposit their feces or their cadavers, bat guano being a prime example, (2) streams flowing into caves that can bring dissolved organic carbon, but also particulate organic matter, sometimes of considerably large size, and (3) percolating water charged with a variety of compounds containing organic carbon, microbes, soil particles and micro-arthropods. These energy sources are both spatially and temporally variable [7]. Also of importance, *A. mexicanus* cavefish have almost no predators, except for crayfish in some caves. In sum, each cave should probably be considered as a special case, and comparisons between different caves are probably as varied as the comparison between caves and rivers.

Not much is known yet on the general ecological and environmental conditions in which wild *A. mexicanus* cavefish live. For example, it is not known how, when or how often they breed. There is no report on their health condition or parasitic load. Data on their population size, density or demography are very scarce. The seasonal variations they are subjected to are poorly understood. Only their feeding habits have started to be investigated [8]. Analyzing stomach contents in cavefish of the Pachón cave showed that juveniles feed mainly on small arthropods, while adults mostly rely on partially decomposed material, guano or detritus from the mud. This study also showed that contrarily to common belief, Pachón cavefish seem relatively well fed. On the other hand, laboratory studies have demonstrated that cavefish originating from the Pachón cave are excellent at finding food. Both at larval [9] and at adult [10] stages, they out-compete their surface fish conspecifics for foraging in the dark. Indeed, while surface fish feeding behavior is mainly visually driven, cavefish have evolved a number of traits that seem advantageous to find food in the dark. They possess more taste buds [11, 12], more neuromasts [11, 13], and larger nostrils and olfactory epithelia [14, 15] than surface fish. These sensory specializations are coupled to a newly evolved vibration attraction behavior to locate moving objects [13] and an excellent sense of smell to detect low concentrations of food-related odors [14, 15]. Cavefish have also evolved a special feeding posture that is highly efficient for bottom feeding [16]. Finally, metabolic changes have been described in some cavefish populations. A mutation in the melanocortin receptor 4 (Mc4r) increases appetite, growth and starvation resistance in Tinaja cavefish [17], while Pachón cavefish exhibit different energy stores together with a hypometabolism [18]. Cavefish in the wild and in the laboratory store much more fat than their surface conspecifics (personal observations, SR/LE/POG).

Here, a scalometry approach was used in order to globally and comparatively address growth in cavefish and surface fish, and to get insights on the implications of this life history trait on the adaptation of cavefish to their habitat. Three caves considered to be representative of distinct cave environment have been sampled: Pachón, Subterráneo and Tinaja (Fig. 1a). Pachón is a small cave, containing the most studied *A. mexicanus* cavefish population and located in the North of the Sierra de El Abra, on its western slope. The cave entrance is at an elevation of 210 m [1]; hence, Pachón is considered as a “perched” and isolated cave, and there is no stream of water entering it. The cave hosts a small bat colony. Tinaja on the other hand is a great cave located in the southern half of the Sierra de El Abra. Its entrance, at the base of the Sierra, lies at the end of an impressive 53-m-deep canyon. The river bed in the canyon was dry in March when we visited the area (end of the dry season), but it carries flowing water during the rainy season. We collected scales from fish in the first, muddy pool, located 425 m from the entrance, called the “Traverse Lake” [1]. Percolating water is very abundant in this pool. Finally, the Subterráneo cave is located in the Micos area, with its entrance being at the base of the Sierra de Colmena [1], in a polje. The cavefish population hosted there is independently evolved from those found in the caves of the Sierra de El Abra [19]. During the rainy season, the Subterráneo cave receives enormous influxes of running water carrying trees and sugar canes. These water flows also carry surface fish populating local streams (*Astyanax* and other species), which are washed inside the cave, where they cohabit with resident cavefish and sometimes breed. This results in individuals with a hybrid, F2-like phenotype [14]. There are crayfish in this cave, and we have personally observed predation on cavefish (SR, LE). In this study, age/size relationships were compared among caves, between caves and nearby rivers, and also between wild fish and laboratory-raised fish.

## Methods

### Wild samples

In this paper, within the species *A. mexicanus*, “surface fish” is used to refer to the eyed and pigmented fish living in or originating from rivers, while “cavefish” refers to troglomorphic (blind and depigmented) morphs living in or originating from caves. Then, the exact origin (the name of the rivers for surface fish and the name of the cave for cavefish) is also given.

Scales were sampled during two field expeditions in the state of San Luis Potosi and Tamaulipas, Mexico, in March and November 2016, under the auspices of the collecting permit 02438/16, delivered by the Mexican Secretaria de Medio Ambiente y Recursos Naturales to POG and SR. Three caves hosting *A. mexicanus* troglomorphic cavefish populations were visited (Fig. 1a): Pachón (near the village of Praxedis Guerrero; scales collected from *n* = 69 fish), Tinaja (near the village of El Sabino; *n* = 12) and Subterráneo (near the village of Los Otates; *n* = 26). *A. mexicanus* surface fish were sampled in the Rio Gallinas close to the village of Rascon (*n* = 44) and in the Arroyo Lagarto (*n* = 39). Total length (TL) of all individuals (cm) was determined, and water temperature was recorded at all sampling points during these expeditions as well as previous expeditions (Table 1).

**Table 1:** Sampling in various cavefish and surface fish localities, as well as in the laboratory facility, to deduce age/size relationships

Origin	Population	Number of fish sampled	Number of fish analyzed	TL (cm) min–max	Age (months) min–max	Water temperature
Caves, wild-caught	Pachón cavefish	69	55	2.6–6.5	24–60	24.3–24.5 °C
	Tinaja cavefish	12	12	3.0–8.5	36–108	23.2 °C
	Subterráneo cavefish	26	13	4.0–7.5	36–60	23.7–23.7 °C
Rivers, wild-caught	Arroyo Lagarto surface fish	39	37	3.5–7.9	36–72	25 °C
	Rio Gallinas surface fish	44	42	2.8–6.1	24–60	25–25.3 °C
Laboratory-reared	Texas Surface fish	227	227	0.5–9.5	1–89	26 °C
	Pachón cavefish	165	165	0.6–10.0	1–88	23 °C

The fish were collected with dip nets (cavefish) or a seine net (surface fish). Each fish was handled carefully and placed on a Plexiglas plate. Three or four scales were rapidly taken with soft forceps under the pectoral fin and immersed in a tube containing ethanol. Each individual was then individually photographed in a small aquarium containing a ruler for subsequent size measurement (TL) and phenotypic examination (Fig. 1b–f). Specimens were then immediately released in their natural pool.

In addition, 33 surface individuals and 1 Pachón individual were killed by decapitation and immersed in ethanol. Back in the laboratory, the otoliths were dissected out and processed for age analysis in parallel to the scales, in order to corroborate and ascertain the measurements performed on the scales.

### Laboratory samples

Laboratory stocks of *A. mexicanus* surface fish (origin: San Salomon spring, Texas, USA) and cavefish (Pachón population) were obtained in 2004 from the Jeffery laboratory at the University of Maryland, College Park, MD. The colonies in the Gif sur Yvette facility were maintained as described in Elipot et al. [20]. Briefly, for both morphs, embryos and larvae up to 1 month were kept at 23 °C in an incubator and fed twice a day with *Artemia* nauplii. From 1 month to 1 year, they were maintained in nurseries (1.5 L) then in larger tanks (30–60 L) in the facility on a 12:12 h light:dark cycle, at 24 °C, and fed twice a day with *Artemia* and dry food pellets. Finally, 1-year-old fish were incorporated in the breeding colonies and maintained at 23° (Pachón cavefish) or 26 °C (surface fish) in groups of ~ 25–30 fish in 120-L tanks, and spawning was induced by temperature changes (increasing the temperature for cavefish, decreasing the temperature for surface fish, every week). As growth in ectotherms can be influenced by temperature, we calculated that these housing differences resulted in a 0.62 degree day difference between Pachón cavefish (23.94 degree day) and surface fish (24.56 degree day) for 7-year-old fish, averaged on their entire lifetime.

Animals were treated according to the French and European regulations for handling of animals in research. SR’s authorization for use of animals in research including *Astyanax mexicanus* is 91-116 and the Paris Centre-Sud Ethic Committee protocol authorization number related to this work is 2012-0052. A total of 392 fish (165 Pachón, 227 surface fish) born in the facility, with exact known birthdate (therefore not necessitating scales analyses), were measured for their total length to establish growth curves in laboratory-reared conditions.

### Sclerochronology

Scales were rehydrated and photographed using a Nikon AZ100 microscope or a Leica M165C dissecting microscope under transmitted light. Alternating translucent and opaque bands were visible. It was assumed that growth ring consisted of one opaque and one translucent band. Scales were read for annulus identification, assisted by an image analysis system using the TNPC software for digital processing of calcified structures (http://www.tnpc.fr), and taking into account the scale growth law deduced from the distance between the nucleus and the successive annuli. Each sample was analyzed by two readers to evaluate precision. Precision is defined as the reproducibility of repeated measurements on a given scale, whether or not measurements are accurate [21]. Precision was measured from the average percent error (APE), the percentage agreement (PA) and the coefficient of variation (CV). The formula presented by Beamish and Fournier [22] was used to calculate APE:

$${\text{APE}}_{j} (\% ) = 100*\frac{1}{R}\sum\limits_{{i = 1}}^{R} {\frac{{|x_{{ij}} - x_{j} |}}{{x_{j} }}}$$

where *x*_*ij*_ is the *i*th age determination of the *j*th fish, *x*_*j*_ is the average age calculated for the *j*th fish and *R* is the number of times each fish was aged. CV and PA within 1 year (± 1 year) were proposed by Kimura et al. [23] and Campana et al. [24]:

$${\text{PA}} = \frac{{\sum {\left| {n_{\text{diff}} \le 1} \right|} }}{n}$$

$${\text{CV}}_{j} (\% ) = 100*\frac{{\sqrt {\sum\nolimits_{{i = 1}}^{R} {\frac{{\left( {x_{{ij}} - x_{j} } \right)^{2} }}{{R - 1}}} } }}{{x_{j} }}$$

where *R* is the number of times each fish is aged, *x*_*ij*_ the *i*(th) age determination of the *j*(th) fish, *x*_*j*_ is the mean age calculated for the *j*(th) fish and *n*_diff_ is the difference in age determination between the readings of two readers.

Scales were also measured using Image J (version 1.43u) in their proximal–distal axis. These measures were used to calculate scale size-to-body size (TL) ratios.

### Growth modeling

Growth of *A. mexicanus* was tested from different growth models including:

- the von Bertalanffy model [25] without constraint:
  $${\text{TL}}_{t} = {\text{TL}}_{\infty } *\left( {1 - e^{{ - K(t - t_{0} )}} } \right)$$
- the von Bertalanffy model forced *t*_0_ = 0:
  $${\text{TL}}_{t} = {\text{TL}}_{\infty } - \left( {{\text{TL}}_{\infty } *e^{ - Kt} } \right)$$
- the von Bertalanffy model forced TL_1_ = 2 cm:
  $${\text{TL}}_{t} = {\text{TL}}_{\infty } - \left( {{\text{TL}}_{\infty } - {\text{TL}}_{1} } \right)*e^{ - K(t - 1)}$$

where TL_*t*_ and TL_∞_ are, respectively, the total length at age t and the asymptotic total length, *K* the rate at which the asymptote is reached and *t*_0_ the theoretical age (in years) at zero length. The *t*_0_ value has no biological significance. The optimal growth model was identified using the smallest Akaike Information Criterion (AIC; [26]). The AIC balances the trade-off between the quality of fit and the number of parameters used and is defined as:

$${\text{AIC}} = - \,2{\text{LL}} + 2k$$

where *k* is the total number of parameters (including *σ* ^2^) and − 2LL is two times the negative log-likelihood at its optimum.

Statistical analyses were carried out using the open-source statistical package “R.” Mann–Whitney nonparametric tests were used to compare sizes. Likelihood ratio tests were used to compare the von Bertalanffy growth curves between morphs [27]. Differences were considered significant for *p* < 0.05.

## Results

### Sampling

A total of 190 wild-caught *A. mexicanus* cavefish (*n* = 107) and surface fish (*n* = 83) were sampled for scales in March and November 2016 (Table 1 and Fig. 1a–g). Surface fish total lengths (TL) ranged from 2.8 to 6.1 cm in Rio Gallinas (*n* = 44) and from 3.5 to 7.9 cm in Arroyo Lagarto (*n* = 39) (Fig. 1b). For cavefish, the largest sample came from the Pachón cave (*n* = 69). Forty-nine fish were captured from the main pool and 20 from the small, lateral pool. Their TL ranged from 2.6 to 6.2 cm (Fig. 1c). Among the 12 fish sampled in the Tinaja cave, the TL ranged from 3 to 9 cm (Fig. 1d). In Subterráneo, scales were collected from 15 fish in the “entrance pool,” a small temporary pool located 25 m beneath the entrance (including 10 fish with non-troglomorphic features, Fig. 1e, top right photograph), and from 11 cavefish in the next, permanent pool (called pool2), located 50 m further down after a small pit (Fig. 1f). The TL of the Subterráneo cavefish caught ranged from 4 to 8 cm, while the surface fish from the entrance pool were small, between 3 and 5 cm. The latter were not used in the following analyses, because they represent individuals that were washed inside the cave, and thus are neither representative of the troglomorphic condition nor typical for river-dwelling individuals.

### Scale analysis and scale size

**Fig. 2:**
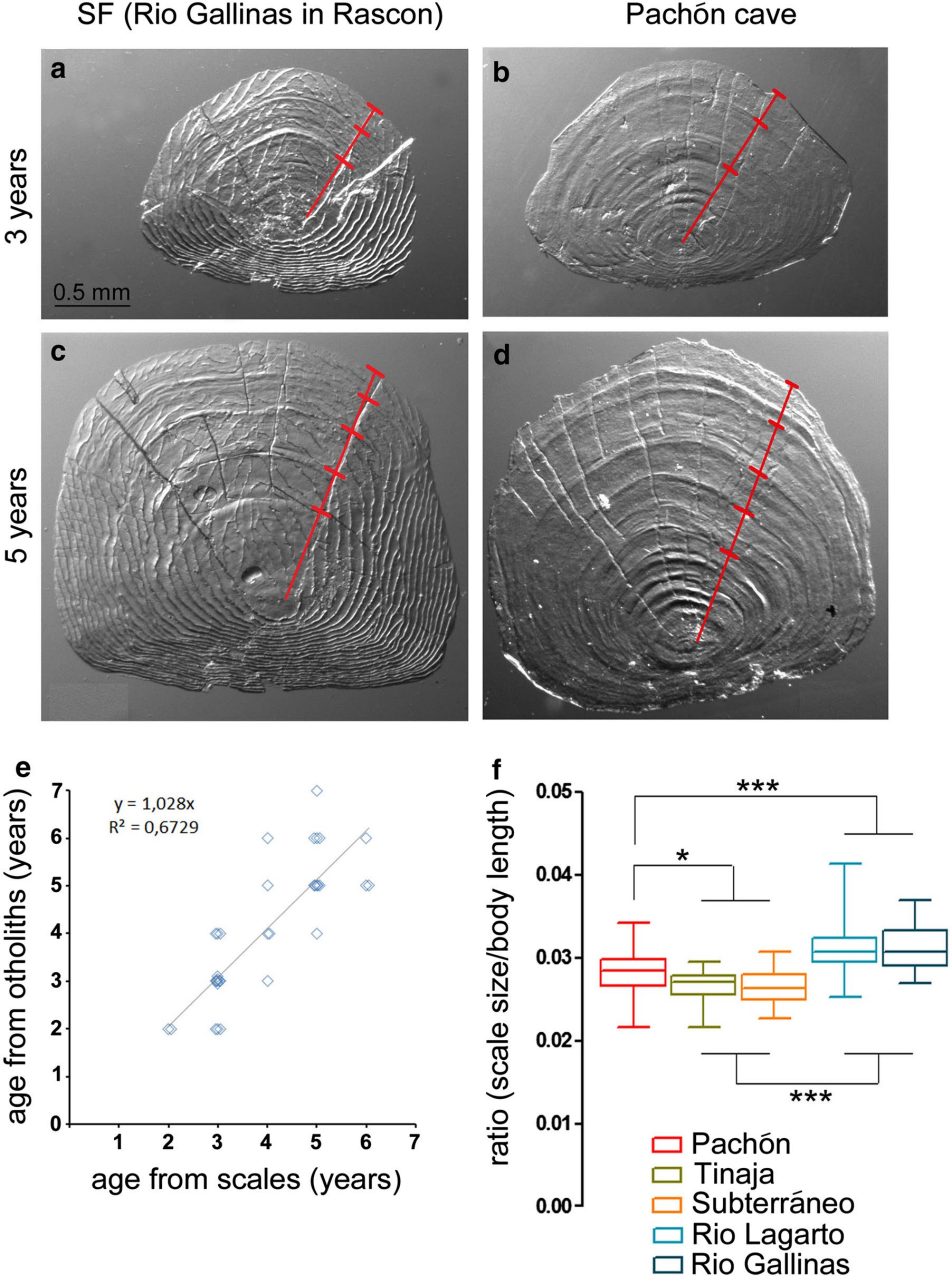
Analyzing scales of *Astyanax mexicanus.* **a**–**d** Photographs of scales of surface fish from Rio Gallinas (left) and from cavefish from the Pachón cave (right). Examples are given for 3-year-old (top) and 5-year-old (bottom) individuals. Annual growth marks are indicated by the red ladder. Scale bar: 0.5 mm. **e** Correlation between age reads from scales and from otoliths for *n* = 33 surface fish individuals. **f** Boxplots showing the scale size/body length (TL) ratios of different *A. mexicanus* populations. The color code is indicated. *p < 0.05 and ***p < 0.0001, Mann–Whitney tests

Scale reading, a sclerochronology method, is a minimally invasive method to infer the age of fishes, which is particularly appropriate in the case of endangered *A. mexicanus* cavefish. This study is the first attempt to deduce the age of an individual from the annuli present on its scales in this species. Importantly, both surface fish and cavefish possessed scales and their scales showed growth marks. Annual growth increments counted on scales were represented by alternations of an opaque and a hyaline zone (Fig. 2). The distance between growth increments decreased from the scale core toward the outer margin. The age bias in the measures, related to reader’s discrepancies, was evaluated, and the results indicated good agreement: The coefficient of variation CV was 3.2%, the percentage of agreement PA was 76.5%, and the average percent error APE was 5.9%. In about 10% of the cases, scales were unreadable. Thus, 158 samples (88.2%) were included in the analysis (Table 1).

In addition, and to strengthen the accuracy of the age reads from scales, scale reads were compared to otolith reads for 1 Pachón individual and 33 surface fish individuals. The otoliths were more difficult to read than the scales. The correlation coefficient between scale readings and otolith readings for a given individual was *R* ^2^ = 0.67 for surface fish (Fig. 2e). In cases of discrepancy, the difference was of 1 year maximum, except for two fish with a 2-year difference. Concerning the single Pachón cavefish analyzed, the age read from scales was identical to the age read from otoliths. Therefore, the age of *Astyanax mexicanus* individuals can be reliably inferred from their scales, both for surface and for cave morphs.

Finally, to document and quantify described differences in the size of scales between the two *A. mexicanus* morphotypes [28], scales were measured in their proximo-distal axis, and individual scale size-to-body size ratios were calculated (Fig. 2f). The three cave morphs had smaller scales than the two surface populations sampled, indicating a significant 10–13% regression of scale size in cavefish. Moreover, Tinaja and Subterráneo cavefish possessed slightly smaller scales than Pachón cavefish, which might suggest that the regression process does not occur at the same pace in independently evolved cave populations.

### Age/size distributions in the sampled locations

**Fig. 3:**
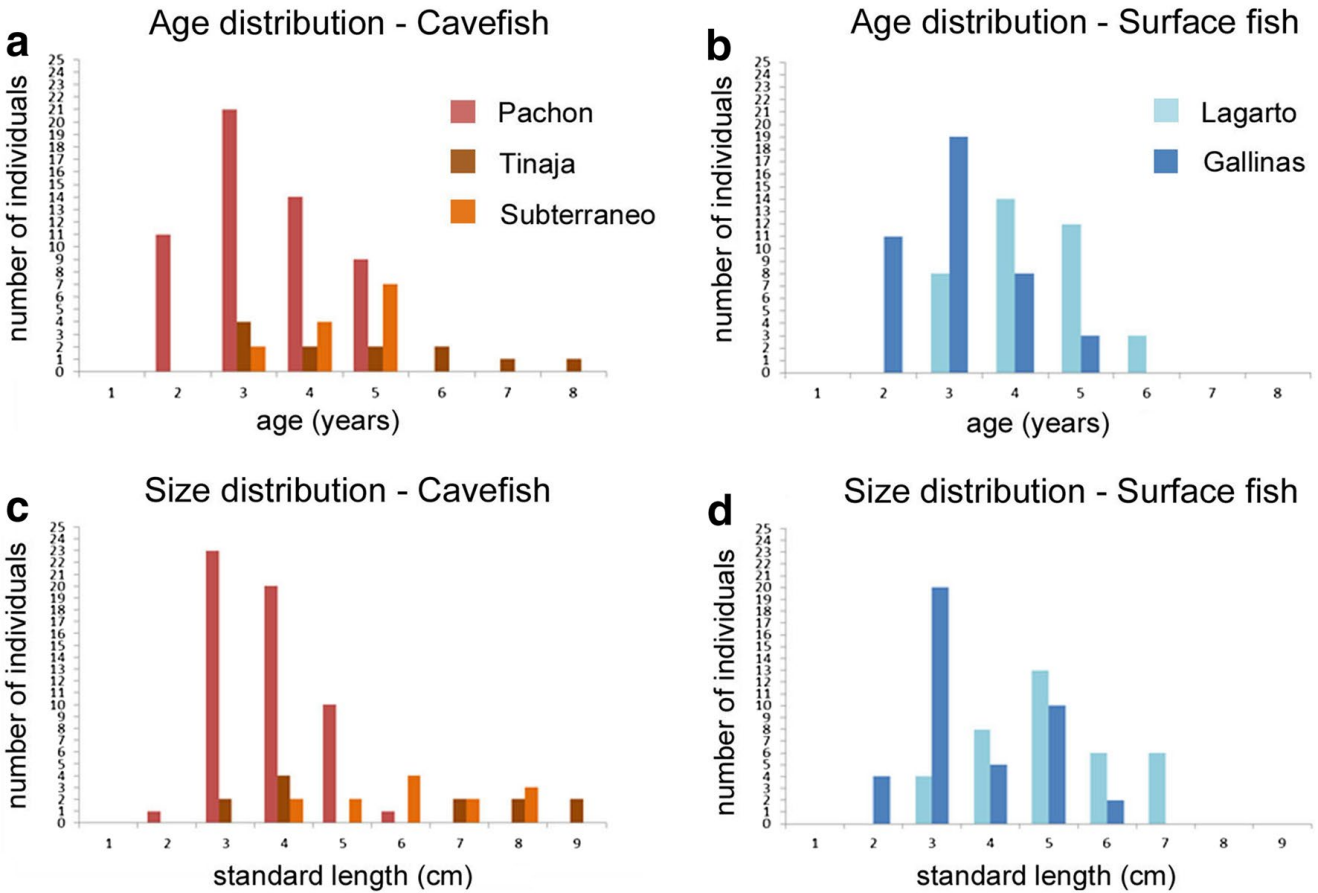
Distributions of sizes (TL) and ages of wild-caught fish. **a**, **b** Age distribution for cavefish (Pachón in red, Tinaja in brown, Subterráneo in orange) and surface fish (Arroyo Lagarto in light blue, Rio Gallinas in blue) caught in the wild and analyzed in this study. **c**, **d** Size (TL) distribution for cavefish and surface fish (same color codes as in **a** and **b**)

The age of the sampled cavefish ranged from 2 to 5 years in Pachón, from 3 to 8 years in Tinaja and from 3 to 5 years in Subterráneo (Fig. 3a). For surface fish, individuals between 2 and 5 years were observed in Rio Gallinas, and fish between 3 and 6 years were found in Arroyo Lagarto (Fig. 3b). These age distributions were very similar to the length distributions found in each location, both for cave and for surface samples (Fig. 3c, d). Moreover, ages deduced from scales increased with TL, and the correlation coefficient between age and size was *R* ^2^ = 0.85 for Pachón cavefish (largest cavefish sample), and *R* ^2^ = 0.69 and *R* ^2^ = 0.66 for surface fish in Rio Gallinas and Arroyo Lagarto, respectively. These results further suggested that scales analyses were accurate.

### Growth of *A. mexicanus* in the wild

**Fig. 4:**
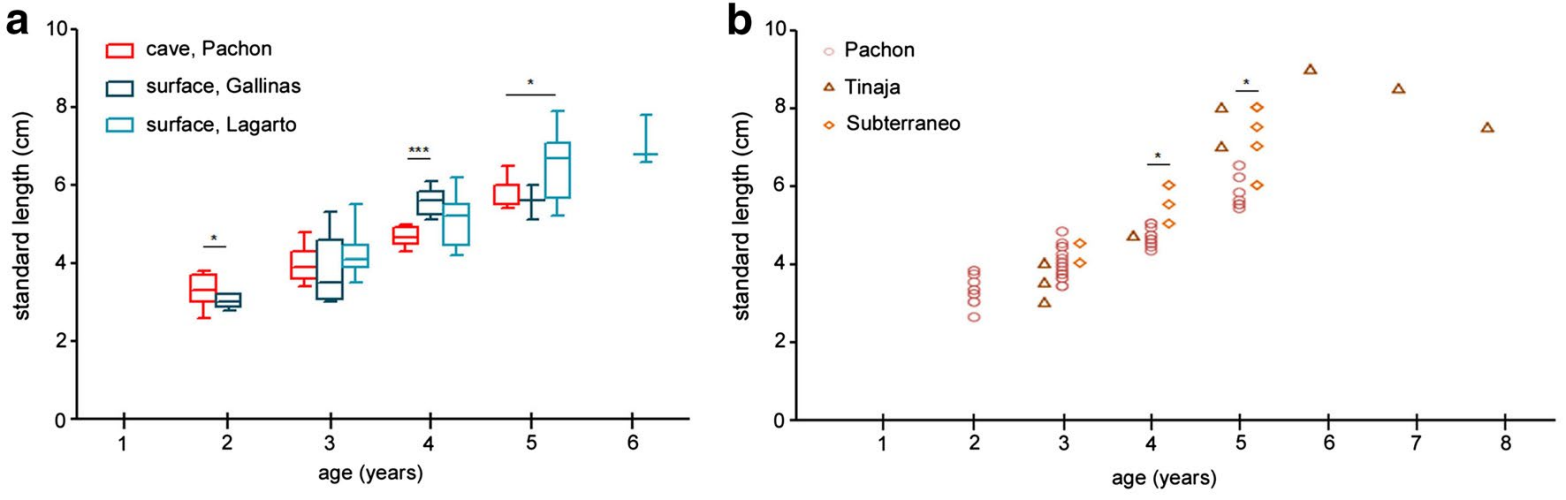
Growth of *Astyanax mexicanus* in the wild. **a** Distributions of sizes (TL) as a function of age compared for Pachón cavefish (red) and surface fish (dark and light blue). *p < 0.05 and ***p < 0.0001, Mann–Whitney tests. **b** Distributions of sizes (TL) as a function of age compared for Pachón (red circles), Tinaja (brown triangles) and Subterráneo (orange squares) cavefish. All sampled fish are shown. *p < 0.05, Mann–Whitney tests

Growth pattern comparisons among the different cave and surface locations are presented in Fig. 4. The growth of surface fish (Rio Gallinas and Arroyo Lagarto) and Pachón cavefish, for which sample sizes are large, is shown in Fig. 4a. Fish showed nonlinear growth. Differences existed between the two morphs. At 2 years of age, Rio Gallinas surface fish were slightly smaller than Pachón cavefish. At 3 years, the Pachón cavefish and the surface fish from the two different river locations had equivalent sizes. Conversely at 4- and 5-year Pachón were smaller than Rio Gallinas and Rio Lagarto surface fish, respectively (Mann–Whitney tests). This may suggest that early growth is relatively faster in the Pachón cave, followed by a growth slowdown at older ages, and that the trend is opposite in rivers. Although several growth models were tested (see “Methods” section), the small sample sizes for young (< 2 years) and old individuals (> 5 years) did not allow to plot growth curves with proper adjustment.

Figure 4b shows comparative growth between caves. For Tinaja and Subterráneo, the sample size was much smaller than for Pachón and did not allow obtaining statistics (*n* = 1 or 2 for several age points) or plotting growth curves. The data are therefore represented with clouds of points for qualitative analysis. Again, fish showed nonlinear growth. From 4 years onward, Tinaja and Subterráneo cavefish were systematically larger than Pachón cavefish at the same age (*p* < 0.05 between Pachón and Subterráneo aged 4 and 5 years old; Mann–Whitney tests). Hence, Tinaja and Subterráneo cavefish older than 4 years old have sizes comparable to surface fish. This may be partly related to the different specific environmental conditions in the three studied caves, as described above. In line, the carbon contents in the mud collected in March 2016 in these three caves (on which adults probably rely, see [8]) were largely variable: 9.2% in the Pachón main pool, 31.9% in the Tinaja Lake and 7.4% in the Subterráneo second pool. Finally, ages greater than 5 years were only found in Tinaja, despite the small sample size from this cave.

### Growth of *A. mexicanus* in laboratory versus wild conditions

**Fig. 5:**
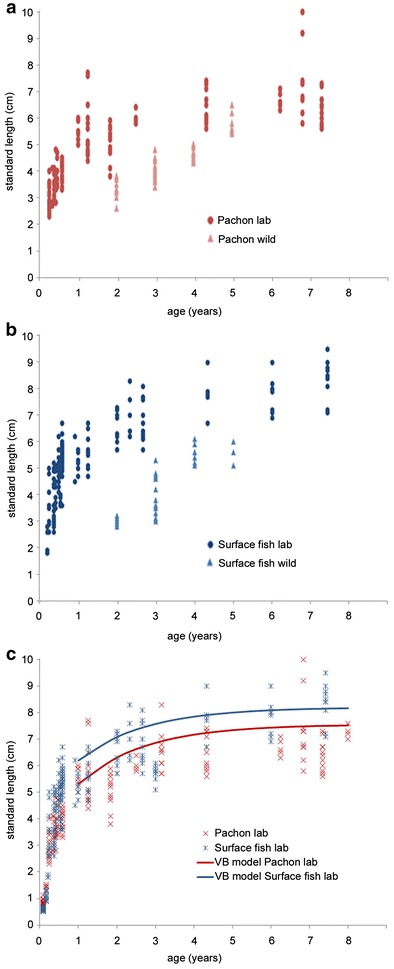
Comparison of growth of *Astyanax mexicanus* in the laboratory and in the wild. **a**, **b** Age/size (TL) relationship of Pachón cavefish in the laboratory (dark red dots) or in the wild (light red triangles; Arroyo Lagarto and Rio Gallinas pooled), and surface fish in the laboratory (dark blue dots) or in the wild (light blue triangles). **c** Superposition of sample distribution (red and blue crosses) and von Bertalanffy modeled growth curves (red and blue lines), for Pachón cavefish and surface fish reared in the laboratory

Finally, to obtain a better view of the impact of food quantity on the growth of the *A. mexicanus* species, age/size distributions were compared between wild-sampled and laboratory-reared animals (Fig. 5). Of note, in the facility, the conditions were not only characterized by food abundance, and other factors such as space, temperature, photoperiod, fish interactions may be determinant for the growth. The laboratory sample was large (*n* = 392; 165 Pachón cavefish and 227 surface fish originating from Texas; Table 1), and fish ages were known exactly, from their birthdates. The laboratory-reared fish showed markedly faster growth patterns (always nonlinear) than wild fish, both for surface and for Pachón morphs. There was almost no overlap of size distribution between laboratory and wild conditions (Fig. 5a, b). In the laboratory, the initial growth was extremely rapid, especially during the first 20–24 months of life. Then, after 2 years, the growth slowed down and size reached a plateau around 8–8.5 cm of total length for surface fish and around 7 cm for Pachón cavefish (see also Fig. 5c). In the laboratory, the largest surface fish measured was 9.5 cm (age 7) and the largest Pachón cavefish sampled was an exceptional 10-cm-long fish (age 6). On the other hand, the largest fish found in Mexican rivers was 7.8 cm (age 6) and the largest fish found in the Pachón cave was 6.5 cm (age 5). Regarding the comparison between growth in the laboratory versus the natural environment (Fig. 5a, b), the same trend was observed for the two morphs, with a higher plateau size in laboratory-reared than in wild-caught individuals, suggesting that surface fish and Pachón cavefish react identically to the food regime. In sum, when they are «overfed» in a laboratory, with the aim of maintaining a breeding colony and rapidly obtaining sexually mature individuals, *A. mexicanus* show a very fast growth, which is significantly different from the more progressive and slower growth observed in the wild.

Finally, with the large samples available from laboratory-reared fish, growth curves fitted with the von Bertalanffy model were plotted (Fig. 5c). The von Bertalanffy model forced TL_1_ = 2 cm was the most precise. A significant difference in length-at-age data derived from this growth model was found between Pachón cavefish and Texas surface fish (likelihood ratio test, *X* ^2^ = 61.79; *p* = 0.018). These data showed that, in identical feeding conditions, surface fish grow faster and larger than Pachón cavefish.

## Discussion

### Scalometry on *Astyanax mexicanus*

This paper is the first study on growth in the species *A. mexicanus*. To our knowledge, it is also the first report on growth on a cavefish species, presenting convergent conclusions from wild-caught and laboratory-reared specimen.

Mark–recapture measures of growth rates on cave Amblyopsids have not confirmed the ages and growth rates originally suggested based on scale marks for these cave fishes [29]. At the start of this study, we were unsure whether *A. mexicanus* cavefish scales would show annual marks, but at the end they were well structured and their interpretation was comparable to those of surface fish scales (contrarily to scales from laboratory-raised fish, which do not show annual growth marks). This indirectly suggests that there might be seasonal variations inside caves, the nature of which will be interesting to document. Indeed, caves are regarded as stable environments, especially in terms of temperature along the year (confirmed by authors’ personal observations and records). However, flash floods or raises in water levels at the rainy season may account for the appearance of annual growth increments in caves. Here, it was possible to obtain age information from scales with a good accuracy, both on the surface and on the cave morphotypes. About 12% of the scales sampled in the wild could not be read, consistent with the idea that tropical fish growth increments are sometimes difficult to analyze.

These scale analyses were possible because troglomorphic *A. mexicanus* have not yet lost their scales, contrarily to some cyprinid species such as the Chinese cavefish *Sinocyclocheilus anshuiensis* which possess only rudimentary scales [30], the scale-less Somalian cavefish *Phreatichthys andruzzii* or the recently discovered first European cavefish, a loach of the genus *Barbatula* that is also scale-less [31]. Wilkens had reported that cavefish scales looked slightly smaller than surface fish scales [28]. We quantified and calculated a 10–13% reduction in scale size in wild cavefish populations when compared to wild surface fish. This reduction was also observed in the laboratory (− 7%; *n* = 47 Texas surface fish, *n* = 48 Pachón cavefish; *p* = 0.0014, Mann–Whitney test), suggesting that the reduction in scale size is not due to food limitations in the natural cave environment. Such a modest reduction may suggest that scale regression has begun and is an ongoing process in *A. mexicanus* cavefishes. Moreover, as Pachón scales seem slightly less reduced than Tinaja or Subterráneo scales (although the sample is small for these two caves), the regression process may not occur at the same pace in different cave populations. As the scale-less *Barbatula* cave loaches have a very recent origin (20,000–16,000 years ago, after the retreat of the last European glaciers [31]—but note that the surface *Barbatula* species have a relatively small number of scales) and the *A. mexicanus* Pachón cavefish are also proposed to have a recent origin about 25,000 years ago [32], the extent of regressive evolution of scales cannot be taken as an index of troglomorphism or “age” of a cave-dwelling species.

The “traditional” von Bertalanffy growth model was the best fitted to the data and allowed tracing satisfactory growth curves with the large dataset obtained from laboratory-reared fish. This same equation has often been used to model the growth of other tropical characiform or related fishes of all sizes: the tambaqui *Colossoma macropomum*, the largest characin of South America which may reach 1 m [33], the red-bellied piranha *Serrasalmus nattereri* of the Amazonas [34], the sister species of *A. mexicanus*, *Astyanax eigenmanniorum* from Argentina [35], or the catfish *Calophysus macropterus* [36], a siluriform that belongs, with characiforms, to the Ostariophyses. Here for wild *A. mexicanus*, the sample was too small at the extremes of the growth curves (very small/young and very large/old), and therefore, it would be worth continuing sampling fish for extreme sizes in future expeditions, to obtain modeled growth curves for wild animals.

### Comparing growth in different *A. mexicanus* morphs and populations

In the wild and in the laboratory, Pachón cavefish seem to reach smaller sizes than surface fish from three different locations: Rio Gallinas and Arroyo Lagarto (wild sampling) and Texas (laboratory-reared population). Although this needs confirmation because some samples are small, Pachón cavefish also seem to grow to smaller sizes than the two other cavefish populations studied, Tinaja and Subterráneo.

Temperatures in the sampled rivers can vary from 22.2 to 28.1 °C, while temperature in the three studied caves is more uniform, between 23.2 and 24.5 °C (data collected from successive expeditions; 2011–2017). In the laboratory, the adult surface fish colony is kept at 26 °C, while the adult Pachón colony is kept at 23 °C (but note that the overall difference in degree day is only 0.62 between the two morphs, as calculated for 7-year-old fish). However, the hypothesis that reduced growth and smaller maximum size in Pachón are due to differences in water temperature can probably be ruled out. First, the temperatures recorded in the three sampled caves were very similar (Table 1) and cannot account for the preliminary differences observed between Pachón cavefish on the one hand and Subterráneo and Tinaja cavefish on the other hand. This between-cave difference would be better explained by variations in terms of energy resources available in the different cave environments and configurations. This possibility is partly supported by the measures of mud carbon content performed in March 2016 (end of dry season), showing that Tinaja mud is much richer than Pachón mud, for example. Second, in the laboratory, where feeding of surface fish and Pachón cavefish is identical and abundant, the von Bertalanffy modeled growth curves are parallel and significantly different, showing that the maximum size reachable by Pachón cavefish is smaller than the maximum size reached by surface fish. Moreover, up to the age of 1 year, Pachón cavefish and surface fish of the laboratory were housed at the same temperature, thus excluding that the difference in size already observable at this age on the von Bertalanffy modeled growth curves can be attributable to differences in water temperature. Thus, there seems to be an intrinsic, probably genetic limitation in growth in Pachón cavefish, the origin of which is currently unknown. This growth limitation may be an advantageous way of limiting energy expenditure and food needs in the cave environment.

Interestingly and contrarily to Pachón, Subterráneo and Tinaja cavefish do not seem to present such a growth limitation. Between 4 and 6 years old, their age/size relationship is comparable to surface fish, as observed from wild-collected samples (the largest wild-caught fish was a Tinaja individual of 8.5 cm, aged 7). This result may seem surprising with regard to the common belief that caves are energy-poor environments. This result shows that some cavefish seem to thrive in their special milieu, find sufficient food and grow at the same pace as their surface conspecifics. There are probably several combined explanations to this finding.

First, the cave environment is probably not as “food-poor” as it may seem. As mentioned in the introduction, various sources of carbon and energy are available inside caves, from several origins. For caves with streams flowing in (as the Tinaja and the Subterráneo caves in the present study), there may be few differences between organic carbon and processing rates in caves and similar-sized surface streams, as demonstrated, for example, in streams of Organ cave (West Virginia, USA) [37]. This aspect has not been investigated in the Mexican caves hosting *Astyanax* cavefish, but the carbon content was high in the Tinaja pool. For the Pachón cave, with no stream flowing in, fish must rely on other sources. We have previously reported that juveniles feed on small arthropods that are abundant in this cave, while adults feed on mud and bat guano [8]. Recently, we have also witnessed an adult Pachón attacking a large, 2.5–3-cm-long cave copepod of the species *Speocirolana pelaezi*, which are numerous at the bottom of the Pachón pool. Finally, the finding in Tinaja cave of two fish, measuring about 8 cm (aged 7 and 8 years), suggests that cavefish are healthy and prosper in their caves and probably reach ages comparable to laboratory individuals (the oldest Pachón cavefish in our laboratory colony is currently an 8.8-cm-long, 11-year-old fish).

Second, the social structure and feeding habits as well as population sizes are different in cave pools or in surface streams. In rivers, very large schools of thousands of individuals must compete for available food, while they beware of predators. In caves, population sizes are low, from a few dozens to a few hundred, in lakes of variable sizes [1, 38]. Cavefish are also different in terms of behavior: They do not school [39, 40], they are non-aggressive and have no hierarchical structure in the group [41–43], and they swim constantly in an isolated manner, which must also increase foraging efficiency.

Third, cavefish are equipped with particularly sensitive sensory systems—except eye and vision of course. Their enhanced lateral line and olfactory systems must be advantageous to detect moving preys and odor plumes, respectively (see “Background” section). In sum, the not-so-scarce availability of food in caves, the absence of strong competition and the exceptional food-finding skills of cavefish probably compensate for the difficulty of feeding in the dark and explain that their growth can be comparable to that of surface conspecifics. Thus, the idea that *A. mexicanus* cavefish are starving in the depth of their caves may have to be revised.

### Comparing growth in wild versus laboratory environment

The comparison between wild-caught and laboratory-raised *A. mexicanus* is also interesting. In the facility, the adult fish had been kept in groups of about 25 individuals in 120-L tanks and they had been fed twice a day. For the two morphs, the growth in the laboratory was strongly accelerated in the first 20 months of life as compared to wild conditions. This suggests that (1) laboratory-reared fish are probably largely overfed, although this does not seem to have deleterious consequences on their health, since they can live up to at least 10–12 years and (2) the existence of a size-regulating mechanism that starts being active around 3–4 years of age and that keeps the size of the animal within the range for the species. Of note, surface fish show space-dependent growth, i.e., reduced growth rates in confined conditions, while Pachón and Tinaja cavefish have lost this trait [44]. The present data thus indicate that the rearing conditions in the laboratory are good and non-stressful for surface fish.

## Conclusion

The control of the growth of an organism involves complex interactions between genes, metabolism, nutrition and environment. Research in the natural milieu is needed to understand how cavefish survive in a situation that is drastically different from their ancestor’s habitat. The present results suggest that some cavefish populations (Pachón) have undergone changes in their growth mode and the control of their final size, while some other populations have not (Tinaja, Subterráneo). Future studies will have to investigate the genetic mechanisms underlying these differences in growth regulation.
